# Effect of Variety and Sex on the Carcass and Meat Quality Traits of Guinea Fowl (*Numida meleagris* L.)

**DOI:** 10.3390/ani12212916

**Published:** 2022-10-25

**Authors:** Katarzyna Śmiecińska, Adrian Stępień, Dorota Kubiak

**Affiliations:** Department of Commodity Science and Processing of Animal Raw Materials, University of Warmia and Mazury in Olsztyn, Oczapowskiego 5, 10-719 Olsztyn, Poland

**Keywords:** guinea fowl, variety, sex, carcass, meat quality

## Abstract

**Simple Summary:**

The guinea fowl is an indigenous African bird species that has spread to all continents due its high adaptive capacity. These birds are cheaper to produce than other poultry species because they are effective foragers and are resistant to most diseases. In some African countries, the guinea fowl is the cheapest type of poultry meat. This species is raised for meat in many countries, including the United States, Canada, France, and Italy. However, the guinea fowl is not widely produced or consumed in Europe. Guinea fowl come in many color varieties and enjoy considerable popularity among amateur breeders in small farms and agritourism farms as a source of meat, eggs, and ornamental feathers. Similarly to pheasant meat, guinea fowl meat has low fat content and high protein content, and it is characterized by high nutritional value. Guinea fowl meat is also highly valued for its unique sensory properties, and it can be a viable alternative to other types of poultry meat. The popularization of guinea fowl farming and raising consumer awareness of the eating quality and sensory attributes of guinea fowl meat could contribute to an increase in its consumption.

**Abstract:**

The aim of this study was to evaluate selected parameters of carcass and meat quality in 16-week-old pearl gray and lavender guinea fowl. The birds were raised in summer and fall, in an extensive system. Until 4 weeks of age, the birds were kept indoors, and from week 5 until slaughter they could use outdoor space enclosed by a fence, adjacent to the building. Guinea fowl were fed complete chicken diets: starter (weeks 0–6), grower (weeks 7–12), and finisher (weeks >13). In comparison with lavender guinea fowl, pearl gray birds had higher live weight at slaughter (*p* = 0.001) and higher carcass weight (*p* = 0.001). Females, compared with males, had a higher carcass dressing percentage (*p* ˂ 0.001), lower liver weight (*p* = 0.008), lower heart weight (*p* ˂ 0.001), and lower total weight of giblets (*p* = 0.004). The leg muscles of pearl gray guinea fowl, compared with those of lavender birds, had a higher content of dry matter (*p* ≤ 0.029). The breast muscles (*p* ˂ 0.001) and leg muscles (*p* = 0.017) of lavender guinea fowl contained less fat than the muscles of pearl gray birds. The breast muscles of females had a higher content of dry matter (*p* = 0.044) and ash (*p* = 0.016), and lower total collagen content (*p* = 0.028) than the breast muscles of males. The leg muscles of females were characterized by a higher pH (*p* ˂ 0.001), and lower cooking loss (*p* = 0.004) and drip loss (*p* = 0.005) than the breast muscles of males. The breast muscles of lavender guinea fowl, compared with those of pearl gray birds, were characterized by a higher chroma value (*p* ˂ 0.001), and a higher contribution of redness (*p* ˂ 0.001) and yellowness (*p* = 0.002), and their leg muscles were lighter in color (*p* ˂ 0.001), with a higher contribution of yellowness (*p* = 0.041) and a higher hue angle (*p* = 0.037). The value of chroma (*p* = 0.004) and the contribution of yellowness (*p* = 0.002) were higher in the breast muscles of females, compared with males. Neither variety nor sex affected (*p* > 0.05) the evaluated sensory properties of guinea fowl meat or the proportions of total SFAs, total UFAs, total MUFAs, and total PUFAs in the intramuscular fat (IMF) of breast and leg muscles. Meat from guinea fowl of both analyzed varieties can be an excellent alternative to more popular types of poultry meat due to its high nutritional value and eating quality.

## 1. Introduction

The guinea fowl belongs to the class Aves, order Galliformes, and family *Numididae*. The *Numididae* family contains four genera, six species, and several dozen subspecies [1]. The helmeted guinea fowl (*Numida meleagris*) is native to the African continent, and it is the only representative of the genus *Numida*. Wild guinea fowl populations colonize Central and East African steppes and the sub-Saharan regions of West Africa, in particular the Guinea Coast [2,3]. This bird species has spread to all continents due its high adaptive capacity. New color varieties of guinea fowl have been developed, and breeding efforts have been made to fully utilize the genetic potential of these birds [4].

In many West African countries, guinea fowl are the second most important source of meat and eggs after chickens [5]. These birds are cheap to produce because they are effective foragers and are resistant to most diseases [6]. According to Sarica et al. [7], the guinea fowl is the cheapest type of poultry meat in some African countries. This species is raised for meat in many countries, including the United States, Canada, France, and Italy [8,9,10,11]. However, the guinea fowl is not widely produced or consumed in Europe [12]. This species is characterized by high phenotypic variation; therefore, guinea fowl come in many color varieties and enjoy considerable popularity among amateur breeders in small farms and agritourism farms as a source of meat, eggs, and ornamental feathers [4]. 

More than ten guinea fowl varieties with differently colored plumage have been developed to date, and the pearl gray (standard) variety that resembles wild guinea fowl is most popular. White, lavender, chocolate, royal purple, coral blue, and pied (two colored) varieties are also reared by amateur breeders [13]. To effectively utilize the genetic potential of this poultry species, guinea fowl broiler lines characterized by superior performance and productivity are selectively bred in France. New varieties are highly suited for industrial farming [14]. 

In comparison with other poultry species, guinea fowl are characterized by a relatively high carcass dressing percentage and high proportions of valuable carcass cuts [4]. Poultry meat, including guinea fowl meat, should be evaluated for processing suitability and consumer preferences. Similarly to pheasant meat, guinea fowl meat has low fat content and high protein content [11], and it is generally regarded as delicate, lean, and nutritious [15]. The quality of guinea fowl carcasses and meat is affected by variety, sex, slaughter age, diet, and management conditions [16]. According to Sarica et al. [7], most studies investigating the quality of guinea fowl meat were conducted on farms where birds ware kept on litter without outdoor access. Guinea fowl can be raised and bred in intensive, semi-intensive, or extensive systems [9].

The few studies investigating the quality of guinea fowl meat indicate that it can be an excellent alternative to other types of poultry meat due to its unique sensory attributes and high nutritional value [17]. The quality of guinea fowl carcasses and meat remains insufficiently researched, and most of the existing studies focus on the pearl gray variety. The present study was undertaken to fill this knowledge gap. The research hypothesis postulates that guinea fowl variety and sex have a significant effect on carcass quality traits, chemical composition, as well as physicochemical and sensory properties of breast and leg muscles. The aim of this study was to evaluate selected parameters of carcass and meat quality in pearl gray and lavender guinea fowl of both sexes.

## 2. Materials and Methods

The study was conducted in accordance with Directive 2010/63/EU on the protection of animals used for scientific purposes [18]. The experimental materials comprised the carcasses of 12 pearl gray guinea fowl and 12 lavender guinea fowl (6 males and 6 females in each group). Guinea fowl were raised on a private farm located in the Region of Kujawy-Pomerania (Poland), in summer and fall, in an extensive system. Until 4 weeks of age, the birds were kept indoors, and from week 5 until slaughter they could use outdoor space enclosed by a fence, adjacent to the building. Indoor and outdoor area per bird was 0.5 m^2^ and 5 m^2^, respectively. Feed and water were available ad libitum throughout the rearing period. The birds were fed commercial complete chicken diets: starter (weeks 0–6), grower (weeks 7–12), and finisher (weeks >13). The composition of diets fed to guinea fowl in different feeding periods is presented in Table 1.

Each bird was weighed individually directly before slaughter (RADWAG WLC 2/A2 electronic scale with an accuracy of 0.01 g; Morawica, Poland). Guinea fowl for research were randomly selected from a group with a similar body weight. Slaughter (at 16 weeks of age) and post-slaughter handling were carried out in accordance with the current meat industry regulations (Council Regulation (EC) No 1099/2009 of 24 September 2009 on the protection of animals at the time of killing) [20]. The carcasses were eviscerated following the removal of head (between the occiput and the atlas) and legs (at the hock), and they were weighed. The gizzard, liver, and heart were cleaned and weighed. Dressing percentage was calculated as the ratio of hot carcass weight to live weight at slaughter, expressed as a percentage. After chilling at a temperature of 4 °C for 24 h, the carcasses were dressed, and breast muscles (fillets and tenderloins) and leg (thigh + drumstick) muscles were removed. Skin and bones were separated, and the muscles were weighed. All measurements were performed with a high-precision electronic scale (above-mentioned).

The proportions of breast and leg muscles in the carcass were determined relative to the average hot carcass weight. Chilled muscles were packaged in drawstring polyethylene bags, placed in isothermal containers, and transported to the laboratory of the Department of Commodity Science and Processing of Animal Raw Materials (University of Warmia and Mazury in Olsztyn). Breast and leg muscles were evaluated 48 h post mortem.

### 2.1. Methods

#### 2.1.1. Chemical Analyses

The content of dry matter (drying at 105 °C to constant weight), total protein (Kjeldahl method, Kjeltec™ 8400 Auto Distillation Unit, FOSS Analytical, Hilleroed, Denmark), crude fat (Soxhlet extraction with diethyl ether as the solvent, Soxtec™ 2050 Auto Fat Extraction System, FOSS Analytical, Hilleroed, Denmark), and minerals as crude ash (incineration at 550 °C to constant weight) in samples of breast and leg muscles was determined by standard AOAC methods [21]. The collagen content of meat was determined based on hydroxyproline content [22], which was converted into total collagen content using a conversion factor of 8.00.

#### 2.1.2. pH

The values of ultimate pH (pH_u_) in breast and leg muscles were measured in meat homogenates (meat to redistilled water ratio of 1:1) with the use of a combination Polilyte Lab electrode (Hamilton Bonaduz AG, Bonaduz, Switzerland) and the 340i pH-meter with a TFK 325 temperature sensor (WTW Wissenschaftlich-Technische Werkstätten, Weilheim, Germany).

#### 2.1.3. Drip Loss

Meat samples weighing around 100 g were cut out from breast and leg muscles after fat and epimysium had been removed. The samples were weighed, a thin line was threaded through each sample, they were placed in tightly sealed plastic drawstring bags so as to prevent contact between the sample and the bag, and hung at a temperature of 4 °C for 48 h. Then the samples were weighed again, and drip loss was calculated as the percentage of their initial weight [23].

#### 2.1.4. Cooking Loss

Meat samples weighing around 140 g were cut out from breast and leg muscles after fat and epimysium had been removed. The samples were weighed and placed in water-tight bags in a water bath so as to prevent their contact, at a temperature of 80 °C for 60 min. Then the samples were cooled to a temperature of 5 °C for 30 min [24], blotted and weighed again. Cooking loss was calculated as the percentage of their initial weight.

#### 2.1.5. Color

Color was determined based on the values of CIELAB coordinates, L* (lightness), a* (redness), b*(yellowness), C* (chroma), and h° (hue angle) [25]. The color space parameters L*, a*, and b* were measured by the reflectance method using the HunterLab MiniScan XE Plus spectrocolorimeter (Hunter Associates Laboratory Inc., Reston, VA, USA) with standard illuminant D65, a 10 standard observer angle (D65/10°) and a 2.54-cm-diameter aperture. Each data point (L*, a*, b*) was the mean of three replicates measured at randomly selected points on the ventral side of left breasts and left legs (two on the thigh and one on the drumstick). Based on the above parameters, the values of chroma and hue angle were calculated as follows: C* = (a*^2^ + b*^2^)^1/2^ and h° = arctan (b*/a*), respectively.

#### 2.1.6. Sensory Analysis

The sensory properties of breast and leg muscles were evaluated after the removal of fat and epimysium. Meat cubes weighing around 50 g were cut out from the muscles across the grain. The samples were heated in 0.6% NaCl solution (solution to sample ratio of 2:1 *v*/*v*) at a temperature of 96 °C for 1 h. The sensory analysis was performed under standard conditions, immediately after heat treatment, by six trained panelists selected for their sensory sensitivity, on a five-point scale (1 points—lowest grade; 5 points–highest grade) [26]. 

#### 2.1.7. Shear Force

Shear force was measured in the INSTRON 5542 universal testing machine (Instron, Canton, Massachusetts, USA) fitted with a Warner-Bratzler head (500 N, speed 100 mm/min). Samples and breast and leg muscles were heated in a water bath at a temperature of 80 °C for 60 min, and then they were cooled at a constant temperature of around 5 °C for 30 min [24]. Cooled samples were dried, wrapped in aluminum foil, and stored at a temperature of 4 °C for 24 h. Next, cylinder-shaped meat subsamples (diameter—1.27 cm, height—2 cm) were cut out from the samples. The maximum shear force required to cut each subsample across the grain was recorded. The final result was the arithmetic mean of three measurements of the maximum shear force in each of the analyzed muscles.

#### 2.1.8. Fat Extraction and Fatty Acid Profile

Intramuscular fat (IMF) was extracted from breast and leg muscles by Soxhlet extraction with diethyl ether as the solvent, in the Soxtec™ Avanti 2050 Auto Fat Extraction System (FOSS Analytical, Hilleroed, Denmark) [21]. Fatty acid methyl esters were prepared by the modified method of Peisker [27] with the use of a chloroform:methanol:sulfuric acid mixture (100:100:1 *v*/*v*). The fatty acid profiles were determined by gas chromatography, using the AGILENT TECHNOLOGIES 6890N (Santa Clara, CA, USA) system with a flame-ionization detector (FID). Samples (1 µL) of fatty acid methyl esters were separated on a capillary column (length: 30 m, inner diameter: 0.32 mm, liquid phase–Stabilwax^®^, film thickness: 0.25 µm) (according to PN-EN ISO 12966-1:2015-01 and PN-EN ISO 12966-2:2017-05). Analyses of samples and reference standards were performed under identical conditions, i.e., carrier gas–helium, carrier gas flow rate 1.5 mL/min, injector temperature 230 °C, detector temperature 250 °C, column temperature 195 °C, dispenser with 1:50 split. The results are expressed as percentages of saturated and unsaturated fatty acids in the total fatty acid pool in intramuscular fat. The fatty acids were divided into the following categories: saturated fatty acids (SFAs), unsaturated fatty acids (UFAs), including monounsaturated fatty acids (MUFAs) and polyunsaturated fatty acids (PUFAs), desirable hypocholesterolemic fatty acids (DFAs) (UFAs + C18:0), undesirable hypercholesterolemic fatty acids (OFAs) (SFAs − C18:0), and EFAs-essential fatty acids (C18:2 + C18:3). The following ratios were calculated: DFA/OFA, UFA/SFA, MUFA/SFA, and PUFA/SFA. 

### 2.2. Statistical Analysis

The results were processed statistically using the STATISTICA program, version 13.3 (TIBCO Software Inc., Palo Alto, CA, USA). The normality of data distribution was checked by the Shapiro–Wilk test. The effects of experimental factors (guinea fowl variety and sex) on the analyzed parameters of breast and leg muscles, and their interactions, were determined by two-way ANOVA. If interactions between the experimental factors were not found (*p* > 0.05), the significance of differences between group means was estimated by Tukey’s test. The significance of differences between group means was determined at *p* ≤ 0.05. Variability was expressed as the standard error of the mean (SEM).

## 3. Results

### 3.1. Carcass Characteristics

Live weight at slaughter, dressing percentage, hot carcass weight, the total weight of giblets, and the proportions of breast and leg muscles in the carcasses of male and female guinea fowl of pearl gray and lavender varieties are presented in Table 2. In comparison with lavender guinea fowl, pearl gray birds had higher live weight at slaughter (*p* = 0.001) and higher hot carcass weight (*p* = 0.001). Sex had no effect on these parameters (*p* = 0.889 and *p* = 0.179, respectively). Dressing percentage was similar in guinea fowl of both varieties (*p* = 0.182), but it was higher (*p* < 0.001) in females than in males. The proportions of breast and leg muscles in the carcass were not affected by variety (*p* = 0.604 and *p* = 0.714, respectively) or sex (*p* = 0.977 and *p* = 0.255, respectively). Variety had no significant effect (*p* > 0.05) on liver weight, heart weight, or the total weight of giblets. However, liver weight (*p* = 0.008), heart weight (*p* ˂ 0.001), and the total weight of giblets (*p* = 0.004) were higher in males than in females. The interactions between variety and sex were not significant (*p* > 0.05) for all parameters presented in Table 2.

### 3.2. Chemical Composition

The chemical composition of breast and leg muscles of male and female guinea fowl of pearl gray and lavender varieties is presented in Table 3. The dry matter content of breast muscles was comparable (*p* = 0.449) in guinea fowl of both varieties, but it was higher (*p* = 0.044) in females than in males. The percentage of total protein in the breast muscles of guinea fowl was not affected by variety (*p* = 0.196) or sex (*p* = 0.651). The fat content of breast muscles was lower (*p* ˂ 0.001) in lavender guinea fowl than in pearl gray birds, but it was not influenced by sex (*p* = 0.888). The percentage of ash was higher (*p* = 0.016) in the breast muscles of females, and the percentage of total collagen was higher (*p* = 0.028) in the breast muscles of males. Variety had no influence on the content of ash (*p* = 0.686) or total collagen (*p* = 0.353) in the breast muscles of birds.

The leg muscles of pearl gray guinea fowl, compared with those of lavender birds, had a higher content of dry matter (*p* ≤ 0.029) and fat (*p* ≤ 0.017) (Table 3). No significant differences were found in the percentage of total protein (*p* = 0.194), ash (*p* = 0.120) or total collagen (*p* = 0.802) in the leg muscles of pearl gray and lavender guinea fowl. Sex had no effect (*p* > 0.05) on the chemical composition of leg muscles. The interactions between variety and sex were not significant (*p* > 0.05) for all parameters presented in Table 3.

### 3.3. Physicochemical Properties

The physicochemical properties of breast and leg muscles of male and female guinea fowl of pearl gray and lavender varieties are presented in Table 4. The values of pH in breast muscles (measured in meat homogenates) were not affected by variety (*p* = 0.219) or sex (*p* = 0.595). Drip loss measured in breast muscles was also similar in guinea fowl of both varieties (*p* = 0.062) and sexes (*p* = 0.594). The breast muscles of lavender guinea fowl were characterized by lower (*p* = 0.021) cooking loss, compared with pearl gray birds, whereas sex had no influence on this parameter (*p* = 0.876). Color lightness (L*) measured in breast muscles was similar in guinea fowl of both varieties and sexes (*p* = 0.825 and *p* = 0.893, respectively). The breast muscles of lavender guinea fowl, compared with those of pearl gray birds, had a higher contribution of redness (a*) (*p* ˂ 0.001) and yellowness (b*) (*p* = 0.002), and a higher chroma value (C*) (*p* ˂ 0.001). Moreover, the breast muscles of females, compared with those of males, had a higher contribution of yellowness (b*) (*p* = 0.002) and a higher chroma value (C*) (*p* = 0.004). The values of hue angle (h°) measured in breast muscles were similar in guinea fowl of both varieties and sexes (*p* = 0.119 and *p* = 0.432, respectively). 

The values of pH measured in leg muscles did not differ significantly between varieties (*p* = 0.428) (Table 4), but they were higher in females than in males (*p* ˂ 0.001). The leg muscles of males were characterized by higher drip loss (*p* = 0.005) and cooking loss (*p* = 0.004) than the leg muscles of females, whereas variety had no significant effect on these parameters (*p* = 0.543 and *p* = 0.072, respectively). The leg muscles of lavender guinea fowl were lighter in color (L*) (*p* ˂ 0.001), with a higher contribution of yellowness (b*) (*p* = 0.041) and a higher hue angle (h^°^) (*p* = 0.037) than the leg muscles of pearl gray birds. Sex had no influence (*p* > 0.05) on the above color parameters. The interactions between variety and sex were not significant (*p* > 0.05) for all parameters presented in Table 4.

### 3.4. Sensory Properties

The sensory properties and shear force values of breast and leg muscles of male and female guinea fowl of pearl gray and lavender varieties are presented in Table 5. Neither variety nor sex affected (*p* > 0.05) the evaluated sensory properties of breast and leg muscles (intensity and desirability of aroma and taste, juiciness, and tenderness on a 5-point scale). It should be noted that the breast and leg muscles of guinea fowl of both varieties received high scores for all sensory attributes. The breast and leg muscles of pearl gray guinea fowl were characterized by lower shear force values than the muscles of lavender birds (*p* = 0.008 and *p* ˂ 0.001, respectively). Sex had no influence on shear force values measured in breast muscles (*p* = 0.806) and leg muscles (*p* = 0.681). The interactions between variety and sex were not significant (*p* > 0.05) for all parameters presented in Table 5.

### 3.5. Fatty Acid Profile

The proportions of SFAs in IMF from the breast and leg muscles of female and male guinea fowl of pearl gray and lavender varieties are presented in Table 6. In comparison with lavender guinea fowl, IMF from the breast muscles of pearl gray birds had a higher content of myristic acid (C14:0) and lower proportions of stearic acid (C18:0) and arachidonic acid (C20:0) (*p* = 0.009, *p* < 0.001 and *p* = 0.011, respectively). The proportions of myristic acid, arachidonic acid and behenic acid (C22:0) were higher in IMF from the breast muscles of males than females (*p* = 0.042, *p* = 0.043 and *p* = 0.010, respectively). Neither variety nor sex affected total SFA concentrations in IMF from breast muscles (*p* = 0.202 and *p* = 0.114, respectively). The proportions of lauric acid (C12:0), pentadecanoic acid (C15:0), palmitic acid (C16:0) and margaric acid (C17:0) in IMF from breast muscles were not affected by variety (*p* = 0.086, *p* = 0.101, *p* = 0.519, and *p* = 0.147, respectively) or sex (*p* = 0.070, *p* = 0.239, *p* = 0.928, and *p* = 0.213, respectively).

In comparison with lavender guinea fowl, IMF from the leg muscles of pearl gray birds had higher proportions of myristic acid and palmitic acid (*p* = 0.011 and *p* = 0.014, respectively), and lower concentrations of stearic acid, arachidic acid, and behenic acid (*p* = 0.006, *p* < 0.001, and *p* = 0.003, respectively). Variety had no effect (*p* > 0.05) on the concentrations of lauric acid, pentadecanoic acid, margaric acid, or total SFAs in IMF from leg muscles. Sex had no influence on the proportions of individual (*p* > 0.05) or total SFAs (*p* = 0.321) in IMF from leg muscles. The interactions between variety and sex were not significant (*p* > 0.05) for all parameters presented in Table 6.

The proportions of MUFAs in IMF from the breast and leg muscles of female and male guinea fowl of pearl gray and lavender varieties are presented in Table 7. The proportions of myristoleic acid (C14:1), palmitoleic acid (C16:1), margaroleic acid (C17:1), oleic acid (C18:1), gadoleic acid (C20:1), and total MUFAs in IMF from breast muscles were not affected by variety (*p* > 0.05), and the proportions of these acids in IMF from leg muscles were not influenced by sex, either (*p* > 0.05). The concentration of gadoleic acid was higher (*p* = 0.018) in IMF from the breast muscles of males, compared with females. The interactions between variety and sex were not significant (*p* > 0.05) for all parameters presented in Table 7.

The proportions of PUFAs in IMF from the breast and leg muscles of female and male guinea fowl of pearl gray and lavender varieties are presented in Table 8. Intramuscular fat from the breast muscles of lavender guinea fowl, compared with pearl gray birds, had higher proportions of γ-linolenic acid (C18:3 n-6), eicosadienoic acid (C20:2), eicosatrienoic acid (C20:3 n-6), docosapentaenoic acid (C22:5 n-3-DPA) and docosahexaenoic acid (C22:6 n-3-DHA) (*p* = 0.005, *p* = 0.027, *p* = 0.010, *p* = 0.007 and *p* = 0.007, respectively), and a lower content of arachidonic acid (C20:4 n-6) (*p* = 0.002). Intramuscular fat from the breast muscles of females, compared with males, had higher proportions of linoleic acid (C18:2), linolenic acid (C18:3), eicosatrienoic acid, DPA and DHA (*p* = 0.010, *p* = 0.005, *p* = 0.012, *p* = 0.023 and *p* = 0.021, respectively), and a lower content of arachidonic acid (*p* = 0.020). Neither variety nor sex affected total PUFA concentrations in IMF from breast muscles (*p* = 0.629 and *p* = 0.414, respectively). 

Intramuscular fat from the leg muscles of pearl gray guinea fowl, compared with lavender birds, had higher proportions of linolenic acid and eicosadienoic acid (*p* = 0.028 and *p* = 0.037, respectively), and lower proportions of eicosatrienoic acid, arachidonic acid, DPA, and DHA (*p* = 0.013, *p* = 0.005, *p* = 0.010, and *p* = 0.003, respectively). Intramuscular fat from the leg muscles of males, compared with females, had higher proportions of γ-linolenic acid, eicosadienoic acid, and arachidonic acid (*p* = 0.048, *p* = 0.004, and *p* = 0.007). Neither variety nor sex affected total PUFA concentrations in IMF from leg muscles (*p* = 0.473 and *p* = 0.601, respectively). The interactions between variety and sex were not significant (*p* > 0.05) for all parameters presented in Table 8.

The fatty acid profile of IMF from the breast and leg muscles of female and male guinea fowl of pearl gray and lavender varieties is presented in Table 9. Intramuscular fat from the breast muscles of pearl gray guinea fowl, compared with lavender birds, was characterized by a higher DFA/OFA ratio (*p* = 0.005). Intramuscular fat from the breast muscles of males, compared with females, was characterized by a higher DFA/OFA ratio (*p* = 0.004) and a lower proportion of EFA (*p* = 0.009). Neither variety nor sex affected the proportions of UFAs, DFAs and OFAs or UFA/SFA, MUFA/SFA and PUFA/SFA ratios in IMF from breast muscles (*p* > 0.05). Variety had no influence on EFA concentrations in the analyzed muscles (*p* = 0.178).

Intramuscular fat from the leg muscles of lavender guinea fowl, compared with pearl gray birds, had higher DFA content (*p* = 0.014), a higher DFA/OFA ratio (*p* = 0.014), and lower OFA content (*p* = 0.014). Variety had no effect on the proportions of UFA and EFA or UFA/SFA, MUFA/SFA, and PUFA/SFA ratios (*p* > 0.05) in IMF from leg muscles, whereas sex had no influence on any of the parameters presented in Table 9 (p > 0.05). The interactions between variety and sex were not significant (*p* > 0.05) for all parameters presented in Table 9.

## 4. Discussion

### 4.1. Carcass Characteristics

Live weight before slaughter, carcass weight, dressing percentage, and the weight and proportion of muscles (in particular breast and leg muscles) in the carcass are the most important indicators of carcass quality in guinea fowl [9]. Similarly to the present study, Kokoszyński et al. [28] and Bernacki et al. [29] found that carcass dressing percentage was higher in females than males, but the differences in the mean values of this parameter were not significant (*p* > 0.05) in the cited studies. Previous research has shown that in poultry, the higher dressing percentage of females results from a lower percentage of giblets and offal [30]. In a study by Kokoszyński et al. [28], gray pearl guinea fowl slaughtered at 16 weeks of age had lower live weight at slaughter (males—1350 g, females = 1377 g), lower carcass weight (males—947 g, females—973 g), and a similar dressing percentage (males—70.1%, females—70.7%) relative to the values noted in this experiment. Das et al. [15] compared three color varieties of guinea fowl and found that at 20 weeks of age, lavender guinea fowl had the highest (*p* ≤ 0.05) body weight (1549.7 g), followed by white (1403.7 g) and pearl gray (1353.2 g) birds. Bernacki et al. [29] demonstrated that live weight at slaughter (*p* ≤ 0.05) was higher in 14-week-old pearl gray guinea fowl (1302 g) than in white birds (1270 g), whereas their dressing percentage was similar (approx. 70%). According to Pudyszak et al. [31], the dressing percentage of guinea fowl was not affected (*p* > 0.05) by age at slaughter (14, 16, and 18 weeks), and it reached 73.61%, 74.49%, and 74.22%, respectively.

In the work of Bernacki et al. [32], the proportions of breast and leg muscles in the carcasses of pearl gray guinea fowl slaughtered at 14 weeks of age were 27.54% and 23.34%, respectively. The above authors also reported that the proportion of leg muscles was higher (*p* ≤ 0.05) in pearl gray than in white birds. In another study by Bernacki et al. [29], the proportion of breast muscles was higher (*p* ≤ 0.05) in females than in males, but only in the white variety. In comparison with the values noted in this study, Pudyszak et al. [31] observed lower proportions of breast and leg muscles (17.66% and 16.36%, respectively) in the carcasses of 16-week-old guinea fowl. In the cited study, the proportion of muscles in the carcass was affected by slaughter age, and their percentage was highest (*p* ≤ 0.01) in guinea fowl slaughtered at 14 weeks of age. The carcasses of guinea fowl evaluated by Zelleke et al. [33] contained 21.4% breast muscles and 20.7% leg muscles. The cited authors also demonstrated that the proportion of breast muscles in the carcass was higher (*p* ˂ 0.0001) in guinea fowl than in chickens of three different genotypes. Yamak et al. [34] compared two production systems and found that the proportion of breast muscles in the carcass was high in both guinea fowl reared in a free-range system (30.84%) and birds reared indoors, in a barn (31.96%). In the cited study, neither gender nor farming system significantly (*p* > 0.01) affected the proportion of breast muscles in the carcass. However, this parameter was significantly (*p* ˂ 0.01) influenced by slaughter age, and it was determined at 30.73%, 31.48%, and 32.12% in guinea fowl slaughtered at 14, 16, and 18 weeks of age, respectively. According to Baéza et al. [35], carcass tissue composition can be affected by the live body weight of guinea fowl (slaughter age) as well as variety and sex. Similar observations were made in the studies cited above.

Kgakole et al. [36] analyzed the weight of giblets (gizzard, heart, and liver) in lavender, pearl gray, and royal purple guinea fowl slaughtered at 20 weeks of age and found that these parameters were not affected by sex or variety (*p* > 0.05). The weight of giblets in the above study was higher than that noted in this experiment, which could result from differences in slaughter age. Zelleke et al. [33] reported lower gizzard weight (22.4 g) and similar liver weight (17.1 g) and heart weight (6.7 g) in guinea fowl slaughtered at 20 weeks of age, relative to the values determined in the present study. In the work of Bernacki et al. [32], the total weight of giblets in white and pearl gray guinea fowl (45 g and 46 g, respectively) slaughtered at 14 weeks of age was lower than the values noted in this experiment. In the cited study, the total weight of giblets was not significantly (*p* > 0.05) affected by variety or sex. Flis et al. [37] observed significant (*p* ≤ 0.05) differences in the weight of giblets between pheasant males and females, but this species is characterized by a high level of considerable sexual dimorphism in body weight and, consequently, the weights of internal organs. In the present study, despite similar body weights of male and female guinea fowl, sex had a significant effect on the total weight of giblets. Bawej [38] evaluated 12-week-old pearl gray and 14-week-old white guinea fowl and found that heart weight was higher (*p* ≤ 0.05) in males than in females, which is consistent with the present findings. Musundire et al. [39] noted higher heart weight and gizzard weight in males than in females, but the differences were not significant (*p* > 0.05). In turn, Kasperek et al. [40] reported that green-legged partridge roosters, compared with capons, had higher (*p* ≤ 0.05) heart weight despite significantly lower body weight. 

### 4.2. Chemical Composition

An analysis of the chemical composition of the breast and leg muscles of guinea fowl confirmed that they had high protein content and low fat content [9]. Boz et al. [41] analyzed the chemical composition of breast muscles in 16-week-old guinea fowl, partridges, and pheasants reared in free-range and intensive systems and noted significant differences (*p* ˂ 0.05) between bird species, whereas the effect exerted by the farming system was not significant (*p* > 0.05). In the cited study, the content of dry matter (26.38%), fat (0.26%), and ash (0.99%) in the breast muscles of guinea fowl was similar to the values determined in this experiment, whereas protein content was more than 2% lower. According to Sarica et al. [7], birds raised under free-range conditions are more active than those kept indoors, therefore the former deposit less fat. In comparison with the present results, Yildirim et al. [11] noted a higher content of dry matter (27.59%) and fat (0.68%), and a similar content of protein (25.86%) and ash (1.05%) in the breast muscles of 16-week-old guinea fowl. The above study confirmed that guinea fowl meat is high in protein and low in fat. Pudyszak et al. [31] analyzed the thigh muscles of guinea fowl slaughtered at 16 weeks of age, and found a similar content of protein (21.26%) and ash (1.06%), and higher fat content (5.86%), compared with the values determined in the current experiment. In the cited study, guinea fowl were raised indoors on deep litter, which could contribute to increased fat deposition in their carcasses due to limited physical movement, compared with birds that had access to outdoor space. Boz et al. [41] reported that only the dry matter content of thigh muscles varied depending on guinea fowl’s age (*p* ˂ 0.05), whereas the effect exerted by production system was not significant (*p* > 0.05). In the above study, the thigh muscles of guinea fowl reared in a free-range system had similar dry matter content (24.31%), and a lower content of protein (19.66%), fat (0.66%), and ash (0.86%), compared with the values noted in this experiment. In the work of Biesiada-Drzazga et al. [42], the leg muscles of 20-week-old pheasants raised in an aviary contained more dry matter (26.31%) and protein (23.30%), and somewhat less fat (1.76%) than the muscles of guinea fowl evaluated in the present study.

In the literature, there is scarce information on the collagen content of guinea fowl meat. Since meat from wild animals is known for its high protein content and low fat content [43], guinea fowl meat is often compared with meat from game birds, in particular pheasants [11]. Daszkiewicz and Janiszewski [44] investigated the effect of sex on the quality of meat from farmed pheasants aged 25 weeks and found that collagen content was higher (*p* = 0.030) in the breast muscles of males than females (0.20% vs. 0.14%). However, sex had no influence (*p* > 0.05) on the remaining chemical components of pheasant meat. An analysis performed by Franco and Lorenzo [45] revealed that the collagen content of drumstick muscles in 10-month-old male pheasants reared in an extensive system was approximately three-fold lower (0.43%) than that determined in this study. According to Gerrard et al. [46], collagen synthesis can be stimulated by testosterone, which explains why collagen content was higher in the breast muscles of males than females in the current experiment.

### 4.3. Physicochemical Properties

In the present study, sex had no effect on the pH of breast or leg muscles in pearl gray and lavender guinea fowl, which is consistent with the findings of Kokoszyński et al. [28] and Nikolova et al. [5]. The pH values measured in breast muscles were not indicative of quality defects such as PSE (pale, soft, exudative) meat. Nikolova et al. [5] demonstrated that neither sex nor age affected (*p* > 0.05) the pH of breast muscles in guinea fowl, measured 25 min, 4 h and 24 h post mortem. Batkowska et al. [4] and Zelleke et al. [33] found that pH values were higher in leg muscles than in breast muscles, which was also observed in the current study. Previous research conducted by Kiessling [47] has shown that the lower pH of breast muscles, compared with leg muscles, results from the type and number of muscle fibers. In addition, the higher pH of leg muscles may be associated with free-range farming. The leg muscles of birds raised under extensive conditions, compared with their counterparts kept indoors, have higher pH values due to lower glycogen content resulting from movement and exercise.

Water-holding capacity is an important indicator of meat quality [5] and processing suitability. In comparison with the present study, Batkowska et al. [4] observed lower drip loss (1.55–1.94%) and cooking loss (15.79–16.95%) in the breast muscles of guinea fowl, and similar drip loss and around 10% lower cooking loss in leg muscles. The cooking loss noted in the breast muscles of guinea fowl (15.52%) by Mohamed et al. [17] was lower than that determined in this experiment. Similarly to the present study, Dahouda et al. [48] reported that sex had no effect on breast muscle drip loss or cooking loss in Benin local guinea fowl. Sarica et al. [7] found that production system (free-range and barn) had no influence on the drip loss or cooking loss of thigh meat in guinea fowl. The lower cooking loss of breast meat, observed in lavender guinea fowl in the present study (as compared with pearl gray birds), could be due to the significantly lower IMF content of their muscles. 

In the work of Musundire et al. [39], meat from female guinea fowl had higher IMF content and therefore was characterized by higher cooking loss, compared with meat from males. According to the above authors, meat loses part of IMF during heat treatment, which contributes to leakage and fluid loss. In the current experiment, an analysis of the effect of sex on drip and cooking losses in the leg muscles of guinea fowl revealed that their values were not affected by fat content, which was comparable in males and females. Moreover, lower drip and cooking losses were noted in the leg muscles of females than males. According to Musundire et al. [39], differences in the drip and cooking losses of meat are affected by heat treatment parameters (temperature and cooking time), poultry species, ultimate pH, and muscle type. In the present study, lower drip and cooking losses in the leg muscles of females, compared with males, could result from a significantly higher pH in the former. Meat drip loss decreases with increasing pH values due to a greater distance between muscle pH and the isoelectric point of myofibrillary proteins [49]. 

Meat color is determined by the content, composition, and transformations of muscle pigments. The changes in the color of guinea fowl meat, observed in this study, were a natural consequence of such transformations. In meat, myoglobin is the major pigment responsible for the red color. Depending on the oxygen partial pressure, dark-red myoglobin can be converted to bright-red oxymyoglobin or brownish metmyoglobin [50]. Myoglobin concentration in muscles varies depending on genetic factors [51], animals’ diet and age, as well as muscle type and pre-mortem activity [52]. The concentration of myoglobin in muscles increases with age, and meat becomes darker red (a*) in color [35]. However, Kokoszyński et al. [28] observed an age-related decrease in the redness of guinea fowl meat, which was attributed to the higher growth rate of birds reared in an intensive system. In the cited study, 16-week-old pearl gray guinea fowl kept indoors without access to outdoor space were characterized by a much darker color (L*) of breast and leg muscles, a higher contribution of redness (a*) and a lower contribution of yellowness (b*), compared with the values determined in the present experiment. In a study by Batkowska et al. [4], meat-type guinea fowl kept on deep litter were characterized by lower values of color parameters L*, a*, and b* in breast muscles, and a lower value of L* and similar values of a* and b* in leg muscles, compared with those determined in this study. In comparison with the present findings, Yildirim et al. [11] noted similar values of a* and b*, and a lower value of L* in the *pectoralis major* muscle of 16-week-old guinea fowl of the standard genotype, raised under an organic system. The differences in L* values could also result from the use of different methods for color analysis [39]. Sarica et al. [7] demonstrated that female guinea fowl had yellower breast meat than males (*p* ˂ 0.01). According to Kokoszyński et al. [28] and Musundire et al. [39], higher fat deposition in females, compared with males, contributes to the yellow tint of their meat. Moreover, Sarica et al. [7] reported that guinea fowl reared in a free-range system had yellower breast meat (*p* ˂ 0.05) than those kept in a barn. It appears that access to green areas and uptake of carotenoids contained in plants may result in higher intensity of yellowness (b*) in meat. In the present experiment, guinea fowl were reared under free-range conditions, which could explain why the value of b* in their muscles was higher than those reported in other studies [4,28] where birds were kept indoor. Due to the lack of prior research on the effect of color varieties of guinea fowl on the analyzed parameters, it is difficult to compare the present results with the findings of other authors.

### 4.4. Sensory Properties

Fadare et al. [53] compared the sensory quality of meat from guinea fowl, ducks, and chickens and found that guinea fowl meat scored highest for overall acceptability. Kokoszyński et al. [28] reported that the sensory properties of breast muscles in 16-week-old guinea fowl were not affected (*p* > 0.05) by sex or age. However, the muscles evaluated in the cited study scored lower for sensory attributes (3.5–3.7 points on a 5-point scale) than those analyzed in the current experiment. Baéza et al. [35] demonstrated that genotype (standard and label) had no significant effect on the sensory properties of roasted breast and thigh meat from guinea fowl. Moreover, in both compared lines, breast and thigh meat from females was more tender than meat from males (*p* ˂ 0.01 and *p* = 0.02, respectively), and thigh meat from females was less intense in flavor than meat from males (*p* = 0.01). Higher meat tenderness may be associated with a lower content or higher solubility of collagen in muscles [54]. However, in the present study, the lower collagen content of breast muscles in female guinea fowl was not correlated with higher tenderness of their meat. Nicolova et al. [5] also found that neither sex nor slaughter age affected the tenderness of *pectoralis superficialis* muscles in guinea fowl (*p* > 0.05). According to Listrat et al. [55], tenderness may be determined by the type and number of muscle fibers, the rate of post mortem proteolytic changes, storage time, ultimate pH, and water-holding capacity. In a study by Musundire et al. [39], sex had no influence on shear force values measured in the breast muscles of guinea fowl, which is consistent with the present findings. The shear force of breast and leg muscles reported for meat-type guinea fowl by Batkowska et al. [4] is similar to the values noted in this experiment. Meat tenderness and juiciness are largely determined by the proportions and diameters of white and red muscle fibers. The diameter of muscle fibers is often correlated with the shear force of meat [29]. Higher values of shear force, which are often observed in older birds [9], may be linked with a higher proportion of mature collagen cross-links [56]. The maximum values of shear force reported in the literature (Warner–Bratzler test) for the muscles of different poultry species vary widely; one of the reasons could be the use of different measurement methods [9].

### 4.5. Fatty Acid Profile

The amount and chemical composition of IMF exert a significant effect on meat quality [57] and nutritional value [58]. The concentrations of fatty acids in meat are determined by various factors, including animal species, breed, carcass fat content, rearing conditions, climatic conditions, and diet [59]. According to Hoffman and Tlhong [60], the differences in the fatty acid profile of meat may result from analytical conditions. According to Domínguez et al. [61], the type of fibers and their cellular metabolism determine the fatty acid composition of muscles. In the work of Bernacki et al. [32], the color variety and sex of guinea fowl had no influence (*p* > 0.05) on the concentrations of myristic, palmitic, and stearic acids or total SFAs in IMF from breast muscles, which were comparable with those noted in the present experiment and reached 43.5% in the white variety and 42.8% in the gray variety. In turn, Batkowska et al. [4] noted a lower proportion of SFAs in IMF from the breast and thigh muscles of guinea fowl raised on deep litter, compared with the values determined in this study. Gálvez et al. [62] demonstrated than the proportions of myristic acid and arachidic acid were higher (*p* ≤ 0.01) in IMF from the breast muscles of male than female turkeys, which is consistent with the results of this study. In the literature, there is no information on the effects exerted by variety and sex on the concentrations of fatty acids in the leg muscles of guinea fowl. In the current study, the SFA content of IMF from leg muscles was not affected by sex, which corroborates the findings of Mieczkowska et al. [63] who analyzed 16-week-old pheasants kept indoors.

According to Stopler [64], in the group of SFAs, myristic acid, palmitic acid, and lauric acid are most hypercholesterolaemic, whereas stearic acid is neutral and has no effect on blood lipoproteins. The above acids contribute to raising cholesterol levels in the blood serum [65] and meat [66]. According to Tufarelli and Laudadio [8], the diet of guinea fowl can be modified to significantly decrease SFA concentrations and increase UFA concentrations. The cited authors replaced soybean meal with dehulled-micronized faba bean in the diet of guinea fowl, which contributed to a decrease in the levels of palmitic acid, stearic acid (*p* < 0.05) and total SFAs (*p* < 0.01) in IMF from breast muscles. 

Bernacki et al. [32] demonstrated that IMF from the breast muscles of white guinea fowl, compared with pearl gray birds, had a higher (*p* ≤ 0.05) content of palmitoleic acid (0.52% vs. 0.40%), which was around four-fold lower than that noted in this study. Similarly to the present experiment, the cited authors found that neither variety nor sex affected (*p* > 0.05) the proportions of margaroleic acid, oleic acid, or total MUFAs in IMF from the breast muscles of guinea fowl. In both varieties, oleic acid was the predominant MUFA in IMF, and similar observations were made in this study. In contrast, Gálvez’a et al. [62] reported that sex influenced the proportion of MUFAs in the IMF of Hybrid turkeys, which was higher in the breast muscles (*p* < 0.05) and leg muscles (*p* < 0.01) of females than males.

Similarly to the current study, Bernacki et al. [32] found that the color variety of guinea fowl affected (*p* ≤ 0.05) the proportions of eicosadienoic acid and DHA in IMF from their breast muscles. However, in the cited study, the sex of birds had no effect (*p* > 0.05) on the proportion of PUFAs in IMF from breast muscles. Rymer and Givens [67] observed that the genotype of broiler chickens affected (*p* = 0.013) the arachidonic acid content of IMF from breast muscles, but it had no effect (*p* > 0.05) on the concentrations of other PUFAs in IMF from breast and leg muscles; the genotype of turkeys had no influence (*p* > 0.05) on the levels of any of the analyzed PUFAs. In the experiment performed by Mieczkowska et al. [63] on 16-week-old pheasants kept indoors, sex had no effect (*p* > 0.05) on the proportions of linoleic, linolenic, and arachidonic acids in breast and leg muscles, which is consistent with the present findings. The concentrations of PUFAs, including n-3 DPA and DHA, in the breast and leg muscles of guinea fowl raised on deep litter in the experiment conducted by Batkowska et al. [4] were lower than those noted in this study. According to Skřivanova et al. [68], dietary vitamin E intake is one of the factors that modify the fatty acid profile of poultry meat. Vitamin E improves fat quality by decreasing the concentrations of SFAs and increasing the levels of PUFAs (in particular n-3 PUFAs). Green fodder is a natural source of vitamin E for birds, therefore its inclusion in the diet of guinea fowl raised in extensive systems may contribute to increasing the PUFA content of meat [69]. Dal Bosco et al. [70] also confirmed that feeding green fodder to poultry can modify the fatty acid profile of meat.

Bernacki et al. [32] found that neither variety nor sex affected (*p* > 0.05) the total concentration of UFAs or the UFA/SFA ratio in IMF from the breast muscles of guinea fowl, which is consistent with the present findings. The values of the above parameters in the cited study and in the current experiment were similar. In the work of Mieczkowska et al. [63], the UFA/SFA ratio was higher (*p* ≤ 0.05) in IMF from the breast muscles of males than females, and the sex of pheasants had no influence (*p* > 0.05) on the PUFA/SFA ratio or the proportion of OFA in IMF from breast and leg muscles. 

## 5. Conclusions

In comparison with lavender guinea fowl, pearl gray birds raised in an extensive system were characterized by higher live weight at slaughter and higher carcass weight, which suggests that they are more suitable for farming. The breast and leg muscles of lavender guinea fowl contained less fat than the muscles of pearl gray birds. Sex had no effect on the chemical composition of leg muscles. The color parameters of leg muscles were influenced by guinea fowl variety, but not sex. Neither variety nor sex affected the evaluated sensory properties of guinea fowl meat or the proportions of total SFAs, total UFAs, total MUFAs, and total PUFAs in IMF from breast and leg muscles. Guinea fowl meat can be an excellent alternative to more popular types of poultry meat. Guinea fowl farming should be developed, and research into this species should be continued due to the high nutritional value of guinea fowl meat, including its high protein content, high concentrations of PUFAs, DFAs and EFAs, and low fat content and a low proportion of OFAs. This study confirmed the high eating quality of meat from both guinea fowl varieties, which could be a valuable component of the human diet. Guinea fowl farming should be popularized, and consumers should be encouraged to choose guinea fowl meat.

## Figures and Tables

**Table 1:** The composition of feed mixture dedicated for guinea fowl depending on the birds’ age.

Item	Age of Birds		
	0–6 Weeks	7–12 Weeks	>13 Weeks
Analyzed nutrients			
Crude protein (%)	19.00	16.50	15.08
Crude fat (%)	4.46	3.84	3.32
Crude fiber (%)	3.70	4.09	4.53
Crude ash (%)	5.70	5.17	4.95
Nutritional value			
Lysine (%)	1.02	0.99	0.79
Methionine + cystine	0.72	0.69	0.61
Threonine	0.63	0.54	0.54
Tryptophan	0.21	0.18	0.18
Calcium	0.92	0.85	0.79
Total phosphorus	0.65	0.57	0.44
Sodium	0.16	0.16	0.16
AME (MJ/kg) *	12.30	11.60	11.50

* AME (Apparent Metabolizable Energy)—calculated according to Polish Feedstuff Analysis Tables (Smulikowska, S.; Rutkowski, A. (Eds.) Recommended Allowances and Nutritive Value of Feedstuffs. In: *Poultry Feeding Standards* (in Polish), 4th ed.; Kielanowski Institute of Animal Physiology and Nutrition, PAS: Jablonna, Poland, 2005) [19].

**Table 2:** Live weight at slaughter, dressing percentage, hot carcass weight, the total weight of giblets, and the proportions of breast and leg muscles in the carcasses of male and female guinea fowl of pearl gray and lavender varieties (means ± SEM).

Trait	Variety		Sex		SEM	*p*-Value	
	Pearl Gray	Lavender	Male	Female		Variety	Sex
Live weight at slaughter (g)	1510.00 ^a^	1424.08 ^b^	1465.00	1469.08	14.198	0.001	0.889
Hot carcass weight (g)	1074.66 ^a^	1001.50 ^b^	1021.58	1054.58	12.131	0.001	0.179
Dressing percentage (%)	71.18	70.29	69.71 ^b^	71.77 ^a^	0.328	0.182	˂0.001
Breast muscles (%)	21.19	21.60	21.41	21.38	0.378	0.604	0.977
Leg muscles (%)	20.61	20.32	20.91	20.03	0.382	0.714	0.255
Liver (g)	18.12	17.48	18.80 ^a^	16.81 ^b^	0.394	0.426	0.008
Heart (g)	6.52	6.11	6.82 ^a^	5.81 ^b^	0.152	0.188	˂0.001
Gizzard (g)	27.99	27.99	28.72	27.25	0.478	1.000	0.127
Total weight of giblets (g)	52.64	51.59	54.35 ^a^	49.88 ^b^	0.834	0.539	0.004

Values within a row followed by different superscript letters, within experimental factors, are significantly different: ^a,b^—*p* ≤ 0.05.

**Table 3:** Basic chemical composition of breast and leg muscles of male and female guinea fowl of pearl gray and lavender varieties (means ± SEM).

Trait	Variety		Sex		SEM	*p*-Value	
	Pearl Gray	Lavender	Male	Female		Variety	Sex
Breast muscles							
Dry matter (%)	26.37	26.19	26.06 ^b^	26.51 ^a^	0.11	0.449	0.044
Total protein (%)	25.15	25.49	25.26	25.38	0.13	0.196	0.651
Fat (%)	0.42 ^a^	0.21 ^b^	0.32	0.31	0.03	˂0.001	0.888
Ash (%)	1.09	1.09	1.08 ^b^	1.11 ^a^	˂0.01	0.686	0.016
Total collagen (%)	0.58	0.53	0.61 ^a^	0.51 ^b^	0.02	0.353	0.028
Leg muscles							
Dry matter (%)	24.86 ^a^	24.35 ^b^	24.56	24.65	0.12	0.029	0.727
Total protein (%)	21.63	21.89	21.78	21.74	0.09	0.194	0.798
Fat (%)	2.39 ^a^	1.98 ^b^	2.18	2.20	0.08	0.017	0.899
Ash (%)	1.13	1.09	1.09	1.12	0.01	0.120	0.366
Total collagen (%)	1.29	1.27	1.32	1.24	0.05	0.802	0.483

Values within a row followed by different superscript letters, within experimental factors, are significantly different: ^a,b^—*p* ≤ 0.05.

**Table 4:** Physicochemical properties of breast and leg muscles of male and female guinea fowl of pearl gray and lavender varieties (means ± SEM).

Trait	Variety		Sex		SEM	*p*-Value	
	Pearl Gray	Lavender	Male	Female		Variety	Sex
Breast muscles							
pH_48h_	5.84	5.92	5.89	5.87	0.03	0.219	0.595
Drip loss (%)	2.75	3.92	3.51	3.15	0.31	0.062	0.594
Cooking loss (%)	22.84 ^a^	21.58 ^b^	22.17	22.26	0.28	0.021	0.876
L* (lightness)	69.60	69.26	69.32	69.54	0.75	0.825	0.893
a* (redness)	5.89 ^b^	7.44 ^a^	6.31	7.02	0.24	˂0.001	0.135
b* (yellowness)	15.26 ^b^	17.71 ^a^	15.28 ^b^	17.69 ^a^	0.43	0.002	0.002
C* (chroma)	16.37 ^b^	19.22 ^a^	16.55 ^b^	19.05 ^a^	0.46	˂0.001	0.004
h° (hue angle)	68.80	67.18	67.57	68.41	0.52	0.119	0.432
Leg muscles							
pH_48h_	6.42	6.48	6.32 ^b^	6.57 ^a^	0.04	0.428	˂0.001
Drip loss (%)	1.05	0.99	1.13 ^a^	0.92 ^b^	0.04	0.543	0.005
Cooking loss (%)	30.96	29.61	31.31 ^a^	29.26 ^b^	0.37	0.072	0.004
L* (lightness)	53.65 ^b^	58.88 ^a^	56.38	56.15	0.84	˂0.001	0.894
a* (redness)	8.26	8.19	7.90	8.56	0.22	0.886	0.133
b* (yellowness)	7.57 ^b^	9.25 ^a^	8.42	8.39	0.42	0.041	0.974
C* (chroma)	11.26	12.43	11.61	12.08	0.37	0.121	0.535
h° (hue angle)	42.21 ^b^	47.89 ^a^	46.34	43.76	1.39	0.037	0.366

Values within a row followed by different superscript letters, within experimental factors, are significantly different: ^a,b^—*p* ≤ 0.05.

**Table 5:** Sensory properties (points) and shear force values (N) of breast and leg muscles of male and female guinea fowl of pearl gray and lavender varieties (means ± SEM).

Trait	Variety		Sex		SEM	*p*-Value	
	Pearl Gray	Lavender	Male	Female		Variety	Sex
Breast muscles							
Aroma–intensity	4.66	4.79	4.71	4.75	0.06	0.363	0.764
Aroma–desirability	4.75	4.83	4.79	4.79	0.05	0.429	1.000
Taste–intensity	4.79	4.83	4.79	4.83	0.05	0.689	0.689
Taste–desirability	4.75	4.79	4.71	4.83	0.05	0.697	0.237
Juiciness	4.83	4.75	4.83	4.75	0.05	0.429	0.429
Tenderness	4.75	4.58	4.71	4.62	0.07	0.253	0.572
Shear force value	13.92 ^b^	19.69 ^a^	16.52	17.10	1.14	0.008	0.806
Leg muscles							
Aroma–intensity	4.83	4.88	4.83	4.87	0.04	0.670	0.670
Aroma–desirability	4.83	4.92	4.87	4.87	0.04	0.367	1.000
Taste–intensity	4.79	4.83	4.83	4.79	0.05	0.689	0.689
Taste–desirability	4.83	4.83	4.83	4.83	0.04	1.000	1.000
Juiciness	4.85	4.75	4.83	4.79	0.05	0.223	0.689
Tenderness	4.62	4.62	4.62	4.62	0.07	1.000	1.000
Shear force value	14.02 ^b^	21.63 ^a^	18.26	17.39	1.01	˂0.001	0.681

Values within a row followed by different superscript letters, within experimental factors, are significantly different: ^a,b^—*p* ≤ 0.05.

**Table 6:** Proportions (%) of saturated fatty acids in the total fatty acid pool in the intramuscular fat (breast and leg muscles) of female and male guinea fowl of pearl gray and lavender varieties (means ± SEM).

Fatty Acid *	Variety		Sex		SEM	*p*-Value	
	Pearl Gray	Lavender	Male	Female		Variety	Sex
Breast muscles							
C12:0 (lauric)	0.40	0.15	0.41	0.14	0.07	0.086	0.070
C14:0 (myristic)	1.27 ^a^	1.00 ^b^	1.25 ^a^	1.03 ^b^	0.05	0.009	0.042
C15:0 (pentadecanoic)	0.24	0.27	0.26	0.24	0.01	0.101	0.239
C16:0 (palmitic)	25.81	25.33	25.54	25.60	0.36	0.519	0.928
C17:0 (margaric)	0.43	0.46	0.46	0.43	0.01	0.147	0.213
C18:0 (stearic)	13.63 ^b^	15.59 ^a^	14.98	14.25	0.29	<0.001	0.225
C20:0 (arachidic)	0.27 ^b^	0.33 ^a^	0.33 ^a^	0.28 ^b^	0.01	0.011	0.043
C22:0 (behenic)	0.43	0.53	0.59 ^a^	0.37 ^b^	0.04	0.274	0.010
Total SFAs	42.49	43.68	43.82	42.35	0.46	0.202	0.114
Leg muscles							
C12:0 (lauric)	0.06	0.05	0.06	0.06	0.01	0.082	0.811
C14:0 (myristic)	1.18 ^a^	1.05 ^b^	1.07	1.16	0.03	0.011	0.093
C15:0 (pentadecanoic)	0.23	0.21	0.21	0.23	0.01	0.169	0.145
C16:0 (palmitic)	26.09 ^a^	24.24 ^b^	24.64	25.68	0.38	0.014	0.182
C17:0 (margaric)	0.36	0.35	0.35	0.37	0.01	0.611	0.471
C18:0 (stearic)	13.05 ^b^	14.24 ^a^	13.94	13.34	0.28	0.006	0.194
C20:0 (arachidic)	0.21 ^b^	0.27 ^a^	0.25	0.23	0.01	<0.001	0.449
C22:0 (behenic)	0.17 ^b^	0.27 ^a^	0.24	0.21	0.02	0.003	0.411
Total SFAs	41.34	40.70	40.75	41.29	0.27	0.243	0.321

Values within a row followed by different superscript letters, within experimental factors, are significantly different: ^a,b^—*p* ≤ 0.05. SFAs—saturated fatty acids. * Common names are given in parentheses.

**Table 7:** Proportions (%) of monounsaturated fatty acids in the total fatty acid pool in the intramuscular fat (breast and leg muscles) of female and male guinea fowl of pearl gray and lavender varieties (means ± SEM).

Fatty Acid *	Variety		Sex		SEM	*p*-Value	
	Pearl Gray	Lavender	Male	Female		Variety	Sex
Breast muscles							
C14:1 (myristoleic)	0.11	0.08	0.08	0.11	0.01	0.073	0.068
C16:1 (palmitoleic)	2.49	2.12	2.19	2.42	0.11	0.094	0.302
C17:1 (margaroleic)	0.14	0.13	0.13	0.14	0.01	0.684	0.319
C18:1 (oleic)	21.43	21.44	21.38	21.48	0.38	0.991	0.898
C20:1 (gadoleic)	0.62	0.55	0.66 ^a^	0.52 ^b^	0.03	0.291	0.018
Total MUFAs	24.79	24.33	24.44	24.68	0.46	0.623	0.801
Leg muscles							
C14:1 (myristoleic)	0.16	0.13	0.14	0.16	0.01	0.169	0.247
C16:1 (palmitoleic)	3.11	2.55	2.69	2.97	0.15	0.062	0.379
C17:1 (margaroleic)	0.14	0.13	0.13	0.14	0.01	0.221	0.364
C18:1 (oleic)	23.97	24.21	24.15	24.03	0.38	0.764	0.879
C20:1 (gadoleic)	0.62	0.68	0.67	0.63	0.02	0.139	0.345
Total MUFAs	28.01	27.71	27.79	27.93	0.50	0.772	0.891

Values within a row followed by different superscript letters, within experimental factors, are significantly different: ^a,b^—*p* ≤ 0.05. MUFAs—monounsaturated fatty acids. * Common names are given in parentheses.

**Table 8:** Proportions (%) of polyunsaturated fatty acids in the total fatty acid pool in the intramuscular fat (breast and leg muscles) of female and male guinea fowl of pearl gray and lavender varieties (means ± SEM).

Fatty Acid *	Variety		Sex		SEM	*p*-Value	
	Pearl Gray	Lavender	Male	Female		Variety	Sex
Breast muscles							
C18:2 (linoleic)	17.12	19.12	16.46 ^b^	19.78 ^a^	0.67	0.141	0.010
C18:3 n-6 (γ-linolenic)	0.22 ^b^	0.26 ^a^	0.25	0.24	0.07	0.005	0.874
C18:3 (linolenic)	0.98	0.89	0.81 ^b^	1.07 ^a^	0.05	0.363	0.005
C20:2 (eicosadienoic)	0.45 ^b^	0.59 ^a^	0.52	0.51	0.03	0.027	0.879
C20:3 n-6 (eicosatrienoic)	0.27 ^b^	0.38 ^a^	0.27 ^b^	0.38 ^a^	0.02	0.010	0.012
C20:4 n-6 (arachidonic)	12.58 ^b^	9.15 ^a^	12.31 ^a^	9.43 ^b^	0.18	0.002	0.020
C22:5 n-3 (docosapentaenoi-DPA)	0.43 ^b^	0.62 ^a^	0.44 ^b^	0.61 ^a^	0.04	0.007	0.023
C22:6 n-3 (docosahexaenoic–DHA)	0.65 ^b^	0.95 ^a^	0.66 ^b^	0.93 ^a^	0.06	0.007	0.021
Total PUFAs	32.72	31.98	31.74	32.97	0.73	0.629	0.414
Leg muscles							
C18:2 (linoleic)	23.95	24.58	24.16	24.41	0.44	0.516	0.784
C18:3 n-6 (γ-linolenic)	0.25	0.24	0.26 ^a^	0.23 ^b^	0.01	0.588	0.048
C18:3 (linolenic)	1.36 ^a^	1.22 ^b^	1.24	1.33	0.03	0.028	0.179
C20:2 (eicosadienoic)	0.46 ^a^	0.32 ^b^	0.48 ^a^	0.30 ^b^	0.03	0.037	0.004
C20:3 n-6 (eicosatrienoic)	0.21 ^b^	0.27 ^a^	0.23	0.24	0.01	0.013	0.854
C20:4 n-6 (arachidonic)	3.78 ^b^	4.12 ^a^	4.33 ^a^	3.57 ^b^	0.11	0.005	0.007
C22:5 n-3 (docosapentaenoic-DPA)	0.27 ^b^	0.35 ^a^	0.32	0.31	0.02	0.010	0.794
C22:6 n-3 (docosahexaenoic–DHA)	0.31 ^b^	0.47 ^a^	0.41	0.37	0.03	0.003	0.556
Total PUFAs	30.64	31.58	31.46	30.77	0.63	0.473	0.601

Values within a row followed by different superscript letters, within experimental factors, are significantly different: ^a,b^—*p* ≤ 0.05. PUFAs—polyunsaturated fatty acids. * Common names are given in parentheses.

**Table 9:** Fatty acids profile of intramuscular fat (% of total fatty acids) in the breast and leg muscles of female and male guinea fowl of pearl gray and lavender varieties (means ± SD).

Fatty Acid Parameter	Variety		Sex		SEM	*p*-Value	
	Pearl Gray	Lavender	Male	Female		Variety	Sex
Breast muscles							
Total UFAs	57.51	56.32	56.18	57.65	0.46	0.202	0.114
UFA/SFA ratio	1.36	1.29	1.29	1.36	0.02	0.179	0.161
MUFA/SFA ratio	0.58	0.51	0.56	0.53	0.02	0.103	0.581
PUFA/SFA ratio	0.78	0.73	0.73	0.78	0.02	0.402	0.327
DFAs (UFAs + C18:0)	71.14	71.92	71.16	71.89	0.37	0.316	0.341
OFAs (SFAs − C18:0)	28.85	28.09	28.83	28.10	0.37	0.317	0.341
DFA/OFA ratio	2.48 ^a^	1.55 ^b^	2.49 ^a^	1.54 ^b^	0.17	0.005	0.004
EFAs (C18:2 + C18:3)	18.33	20.28	17.52 ^b^	21.09 ^a^	0.71	0.178	0.009
Leg muscles							
Total UFAs	58.66	59.29	59.25	58.71	0.27	0.243	0.322
UFA/SFA ratio	1.42	1.46	1.46	1.42	0.02	0.250	0.301
MUFA/SFA ratio	0.68	0.68	0.68	0.68	0.01	0.841	0.789
PUFA/SFA ratio	0.74	0.77	0.77	0.75	0.02	0.393	0.476
DFAs (UFAs + C18:0)	71.71 ^b^	73.53 ^a^	73.19	72.05	0.38	0.014	0.139
OFAs (SFAs − C18:0)	28.28 ^a^	26.46 ^b^	26.81	27.94	0.38	0.014	0.139
DFA/OFA ratio	2.55 ^b^	2.79 ^a^	2.74	2.59	0.05	0.014	0.125
EFAs (C18:2 + C18:3)	25.61	26.04	25.67	25.91	0.46	0.643	0.746

Values within a row followed by different superscript letters, within experimental factors, are significantly different: ^a,b^—*p* ≤ 0.05. UFAs—unsaturated fatty acids (MUFAs + PUFAs); DFAs—hypocholesterolemic fatty acids (UFAs + C18:0); OFAs—hypercholesterolemic fatty acids (SFAs − C18:0); EFAs—essential fatty acids (C18:2 + C18:3).

## Data Availability

Data available on reasonable request.
